# Interdisciplinary Significance of Food-Related Adverse Reactions in Adulthood

**DOI:** 10.3390/nu12123725

**Published:** 2020-12-02

**Authors:** Dóra Solymosi, Miklós Sárdy, Györgyi Pónyai

**Affiliations:** 1 Department of Dermatology, Venereology and Dermatooncology, Semmelweis University, 1085 Budapest, Hungary; sardy.miklos@med.semmelweis-univ.hu (M.S.); ponyai.gyorgyi@med.semmelweis-univ.hu (G.P.); 2 Doctoral School of Clinical Medicine, Semmelweis University, 1085 Budapest, Hungary

**Keywords:** food adverse reaction, food allergy, food intolerance, histamine intolerance, dietary biogenic amines, oral allergy syndrome, allergy to preservatives, urticaria, self-reported, physician-diagnosed

## Abstract

*Background*: Adults frequently interpret food-associated adverse reactions as indicators of a food allergy. However, the public perception of food allergy may differ from a clinician’s point of view. The prevalence of patient-reported food allergy tends to be higher than physician-confirmed cases. Dermatological manifestations (urticaria, pruritus, dermatitis, and edema) are frequently reported by patients. *Objective:* The aim of this study was to describe patient-reported symptoms related to suspected food allergies and particularly to characterize and highlight the volume of patients who visit Budapest allergy clinics with suspected food allergies. *Methods:* In this prospective study, adult (≥18 years) patients were examined at the Allergology Outpatient Unit of the Dept. of Dermatology, Venereology, and Dermatooncology, Semmelweis University, Budapest. The examination included a detailed medical history; physical examination; and when necessary the measurement of allergen-specific serum immunoglobulin E (IgE) levels. *Results:* Data from 501 patients (393 women, 108 men) were analyzed. Intolerance to dietary biogenic amines occurred in 250 cases (250/501, 50%). Oral allergy syndrome was confirmed in 71 patients (71/501, 14%). Allergy to food preservatives was diagnosed in 14 (14/501, 3%) cases by a dermatologist-allergist specialist. Five individuals (5/501, 1%) were diagnosed with IgE-mediated food allergy. In some cases (28/501, 6%), edema-inducing/enhancing side effects of drugs were observed which patients had misattributed to various foods. Among the food groups considered to be provoking factors, the most frequently mentioned were fruits (198/501, 40%), milk/dairy products (174/501, 35%), and nuts/oilseeds (144/501, 29%). Overwhelmingly, urticaria (47%) was the most common dermatological diagnosis, followed by dermatitis (20%) and allergic contact dermatitis (8%). *Conclusion:* Improvement is needed in food allergy, food intolerance, and general nutritional knowledge among the general public. According to our data, perceived/self-reported food allergies were overestimated by adults when compared against physician-confirmed food allergies; however, other diseases potentially responsible for food-related problems were underestimated. The prevalence of oral allergy syndrome was high in the cohort. Intolerance to dietary biogenic amines was common, and although the role of dietary histamine and biogenic amine is not entirely understood in eliciting patients’ symptoms, improvements in complaints were reported during the control visits.

## 1. Introduction

Adverse food reactions can be summarized as abnormal reactions to food consumption or digestion and can affect multiple organ systems. The umbrella term “adverse food reaction” includes both food allergies with an immunological background and non-immune-mediated pseudo-allergies (i.e., food intolerance, aversion, and other food-related reactions). The diagnostic and therapeutic approach is determined by this differentiation based on pathophysiology. Similarities between symptoms and an improvement in symptoms with an avoidant diet may be misleading; certain intolerance reactions or gastrointestinal diseases are thus often erroneously described as food allergies [1,2,3,4,5].

A significant percentage (15–25%) of adults experience unpleasant symptoms related to the consumption of various foods. These symptoms are frequently perceived as manifestations of a food allergy, although food allergy can only be confirmed in 0.1–3.2% of reactions [6]. In the remaining cases, other causes, diseases, and external provoking factors (e.g., drugs such as nonsteroidal anti-inflammatory drugs and beta blockers) are responsible for the symptoms. While questionnaire-sourced figures suggest an increase in the prevalence of food allergies in adults, the number of cases with a medical diagnosis does not support this trend [7,8]. A meta-analysis found that the prevalence of self-reported food allergy was 3–35% compared to 1–10.8% for food allergies diagnosed by a physician and confirmed with a food challenge [9]. According to data from the United States, 19% of adults considered themselves to be food allergic and followed an avoidant diet (either oligo- or multi-allergen-free) to alleviate their complaints without seeking a confirmatory medical examination [10]. The overestimation of the presence of food allergies may lead to unnecessary food restrictions and affect patients’ quality of life. Further, therapeutic efforts based on self-diagnosis without medical or dietary consultation are common in patients reporting adverse food reactions [11,12].

The nutritional consequences of avoidance diets, risk factors, and proper medical management are often overlooked by patients.

A food allergy is a reproducible specific immune response to a food ingredient (allergen)—usually a protein—except in allergies to galactose-α-1,3-galactose (α-Gal), an oligosaccharide. Patients diagnosed with α-Gal syndrome (red meat allergy) express IgE antibodies against the α-Gal epitope which presents in tissues of non-catarrhine primates. Catarrhine primates include gibbons, orangutans, gorillas, chimpanzees, and humans. Sensitization occurs due to tick bites, as α-Gal is present in the glycoproteins of their saliva. *Ixodes ricinus* in Europe, *Amblyomma americanum* in the United States, and *Ixodes holocyclus* in Australia are the tick species associated with α-Gal or red meat syndrome. Clinically, the allergy is manifested by an IgE-mediated delayed hypersensitivity associated with the consumption of red meats (e.g., beef, pork, lamb). Patients may experience symptoms related to drugs that contain gelatin or stearic acid as part of their manufacturing processes [13,14,15].

Food allergies can be classified into IgE-mediated, mixed IgE-, and cell-mediated (food allergy-associated atopic dermatitis, eosinophilic esophagitis, and other eosinophilic disorders), and non-IgE-mediated (food protein-induced enterocolitis syndrome, food protein-induced proctocolitis, food protein enteropathy) groups [16]. As celiac disease is one of the most common autoimmune disorders, it also plays an important role in the differential diagnosis of food-related disorders [17].

Diagnostic methods for food allergies should rely on detailed medical and dietary history [5,18]. Medical histories, allergen-specific IgE antibody tests, skin prick tests, double-blind, placebo-controlled food challenges (DBPCFC), or food elimination are the recommended diagnostic methods [4,14]. In adult outpatient units, allergen-specific IgE antibody tests and skin prick tests are typically used in tandem with anamnestic data to confirm or exclude the diagnosis of food allergy [19]. However, the applicability of the latter to atopic individuals is limited due to increased skin reactivity. The irrelevant positivity of food prick tests is also more common on atopic skin. IgE sensitization results in a reliable diagnosis only if it is interpreted by a physician, as sensitization does not usually correlate with clinical symptoms [14,15,16]. While DBPCFC provides the most reliable diagnosis [20], it is not commonly used in everyday clinical practice due to its complex feasibility and resource requirements (cost, labor, adequate intensive care background). The risk of provoked anaphylaxis with oral food challenges is also not negligible. According to five-year data based on 6377 oral food challenges, anaphylaxis and allergic reactions occurred in 2% and 14% of cases, respectively [21].

Oral allergy syndrome (OAS) is also among the IgE-mediated reactions. OAS is more common in adulthood, with a frequency of 13–58% [22]; in a British and Armenian study, their prevalence was 2% and 13%, respectively [23,24]. The prevalence rates, spectrum of pollens provoking complaints, and frequency of complaints depend on the geographical region and the characteristic flora. For example, in areas where the birch tree is endemic, the proportion of birch-allergic patients who experience OAS can reach 50% to 90% [25]. Sensitization to respiratory allergens is an essential first step in the pathomechanism of this symptom complex (pollen-food cross-reaction). It is thus mainly patients with pollen allergies or seasonal rhinitis who experience OAS symptoms. Birch tree, ragweed, and grass pollen hypersensitivity are typical in patients experiencing OAS. Common symptoms (edema localized in the lips, tongue, or oral cavity; itching and tingling sensations in the oral mucosa) of the contact allergen response that occur when consuming the trigger food (fruits, vegetables, nuts) are due to the structural similarity between pollens and food molecules. IgE antibodies against pollens bind to homologous food allergen structures and induce OAS symptoms when consuming cross-reactive vegetables/fruits/spices. Heat treatment usually eliminates the allergenicity of most allergens causing OAS. A mild course of symptoms is characteristic; however, especially in association with a pollen allergy, the reaction may progress (anaphylaxis) [26,27,28,29].

Food intolerance reactions are non-immunological adverse responses elicited by a food or food ingredient consumed in an amount that is generally ingested without complaints by non-intolerant individuals. These reactions often mimic the manifestation of a food allergy. The pathomechanism and etiology behind food intolerance reactions are varied (enzyme deficiency, pharmacological reactions, food toxin reactions, etc.) [30].

There are few evidence-based biomarkers and standardized tests for diagnosing food intolerance. Intolerance is usually a “rule out” diagnosis, so it should be preceded by a careful examination process. Keeping a food diary may assist patients in objectifying their symptoms. Due to the heterogeneous pathomechanism of intolerance reactions, widely used food intolerance tests are unsuitable for diagnosis, and certain unvalidated tests should be avoided altogether (allergen-specific immunoglobulin G (IgG) tests/immunoglobulin G4 (IgG4), electrodermal tests, hair analysis, iridology, kinesiology, etc.) [30,31].

Psychogenic reactions related to food and meals in general can also be classified as pseudoallergies and should be considered within the differential diagnosis (e.g., aversion, avoidant/restrictive food intake disorder according to the Diagnostic and Statistical Manual of Mental Disorders, 5th Edition (DSM-5)) [32].

If patients attribute all their complaints to food, the actual causes may remain hidden. Emphasis should therefore be placed on a comprehensive examination protocol that is not limited to allergy testing. The aim of this study was to first describe the patient-reported symptoms related to suspected food allergies and to discuss and characterize the volume of patients who visit Budapest allergy clinics with suspected food allergies.

## 2. Methods

The prospective cohort consisted of 501 patients over 18 years of age with self-reported or suspected food allergies. These patients believed that a food allergy was responsible for their complaints and generally identified certain foods or food groups as provocative factors; however, some participants experienced symptoms from food and eating in general, while others did not identify trigger foods but were nonetheless certain that they had a food allergy.

Patients were referred to the Allergology Outpatient Unit of the Department of Dermatology, Venereology, and Dermatooncology, Semmelweis University, Budapest, by general practitioners and other specialists. Patients were examined by an allergist-dermatologist specialist consultant.

A detailed medical history was taken first, along with an examination of any skin lesions currently visible. In addition to general considerations (comorbidities, medications, family history, etc.), an allergy-focused medical history was obtained: whether the food consumed was heat-treated, whether the patient or family had an atopic or other allergic disease, what were the duration and course of the symptoms, and whether trigger factors played a role in the development of symptoms (exercise, infection, nonsteroidal anti-inflammatory drugs or other medications, alcohol).

In the absence of visible skin lesions, other previously experienced symptoms reported by the patient (e.g., stomach pain, headache, etc.) were discussed in detail. The relationship between symptoms and food/meals was then discussed to determine whether the complaints were attributable to food allergies or food intolerances or whether they had developed independently.

In justified cases, in accordance with the anamnesis, serum-specific IgE levels of suspected foods and/or pollens (due to pollen-food cross-reactivity) were tested by immunoblotting with the Polycheck^®^ kits (Hungary Food 20, Hungary Inhalation 20) (Table 1) from Biocheck GmbH (Münster, Germany).

The data in the manuscript are not based on an intervention study, which would have required the research to have a separate code of ethics approval. However, all the patients referred, admitted, or treated at the Department of Dermatology, Venereology, and Dermatooncology, Semmelweis University, have signed an institutional patient consent form confirming that the university may use their anonymized medical results for educational or research purposes. Ethics committee approval was therefore not required for this fully anonymized survey of standard practice.

Microsoft Office Excel 2016 (Microsoft Corporation) was used to process and present the descriptive data. Food

The diagnosis of intolerance to dietary biogenic amines was based on the patient’s medical history; the results of other referrals; and a diet symptom diary, if any. More specifically, histamine intolerance was diagnosed [1,35] if the patient presented with two or more typical signs or symptoms [1,35,36] (Table 2) in their medical history, listed a characteristic range of foods causing the complaints, reported a correlation between the amount of biogenic amine-rich foods consumed and the severity of complaints, and if improvement was reported at the control examination after a six-week biogenic amine/histamine-free or -low diet (Table 3). The diet was compiled by the allergists of the Allergology Outpatient Unit. The severity, duration, and onset of the symptoms were all considered [1,35].

An epicutaneous patch test was performed to detect allergies to food preservatives when patients reported symptoms associated with relevant foods (processed, preserved, smoked, and ready-to-eat foods).

In the absence of skin symptoms, other complaints (such as stomach pain, bloating, and headaches) which patients believed to be signs of food allergies were discussed, and their potential relationship to food was examined. After summarizing all the symptoms and complaints, further medical examinations were indicated.

If neither IgE-mediated food allergy nor the possibility of a cross-reaction was found on the basis of the symptoms and/or medical history, further examinations were initiated. Depending on the complaints, patients were referred to other specialists (gastroenterological and pulmonological consultation, ear-nose-throat, dental, gynecological, and urological examination) to elucidate the background cause.

If the symptoms were medication-related (e.g., angiotensin-converting-enzyme (ACE) inhibitors—edema), the referring physician was advised to prescribe an alternative medication if possible.

In the case of purely psychological complaints (e.g., disgust, aversion), the patients were informed and offered psychological or mental health counseling where necessary.

Examinations and recommendations were made individually; multiple examinations and counseling sessions could be requested for one patient. Patients were recalled for control as a follow-up.

## 3. Results

### 3.1. Participants and Referral Characteristics

Data collection for this prospective study was closed after 501 patients had been examined (Table 4). There were more than three times as many females as male patients, with 393 females and 108 males comprising the patient population. Almost two thirds of the patients were under 50 years old (64%). Among the three youngest age groups, the distribution was spread relatively evenly. The study population’s mean age was 44 years. Most of the respondents (58%) were from Budapest.

### 3.2. Culprit Foods Reported by Patients

**Figure 1:**
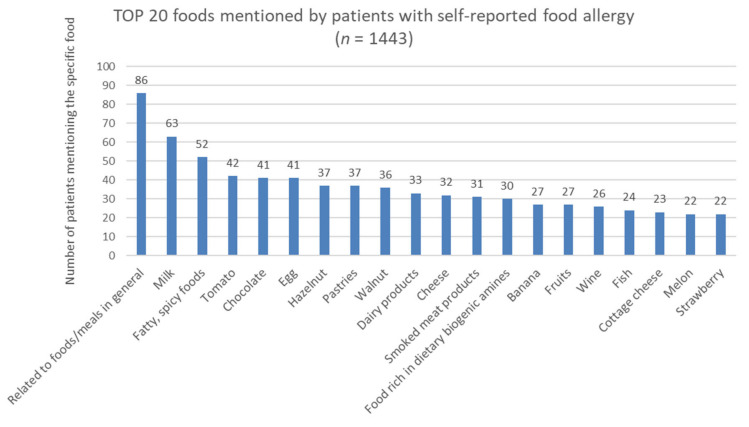
The 20 most commonly mentioned foods in the patient group.

Several foods and food groups were mentioned by patients to have caused an allergic reaction. Patients were allowed to list as many foods as they wished. Altogether, 1443 food records were analyzed. The most common culprit foods are shown in Figure 1. One food record was categorized into one main food group. The culinary classification was used when categorizing foods (e.g., tomato as a vegetable, not a fruit).

Among the food groups considered to be provoking factors, the most frequently mentioned were fruits (198/501, 40%), milk/dairy products (174/501, 35%), and nuts/oilseeds (144/501, 29%). Of the perceived culprit foods, the category “related to foods/meals in general” (86/501) was the most common. In these cases, patients were unable to identify specific foods or food groups responsible for their self-reported food allergies. Milk was listed several times (63/501), after which the categories of fatty or spicy foods and then tomato and chocolate were the most common.

The most important foods rich in dietary biogenic amines are oilseeds (nuts), cocoa, chocolate, hot pepper, paprika, fish and seafood, innards (offal) and smoked meat products, eggs, cheese, fruits (banana, citrus fruits), alcohol, coffee, and sauerkraut. Complaints in relation to these foods were mentioned 484 times (484/1443, 34%) by 332 (332/501, 66%) patients.

### 3.3. Complaints Reported by Patients

The complaints described by patients were diverse. Skin lesions were the most common, but gastrointestinal complaints (abdominal discomfort after eating, bloating, abdominal cramps) as well as shortness of breath, weakness, flushing, and anxiety were also among the reported symptoms. A total of 1426 records were analyzed. The frequency of skin lesions was high (52%, 736/1426), but the percentage (15%, 208/1426) of symptoms characteristic of the involvement of the digestive system was notable as well.

**Figure 2:**
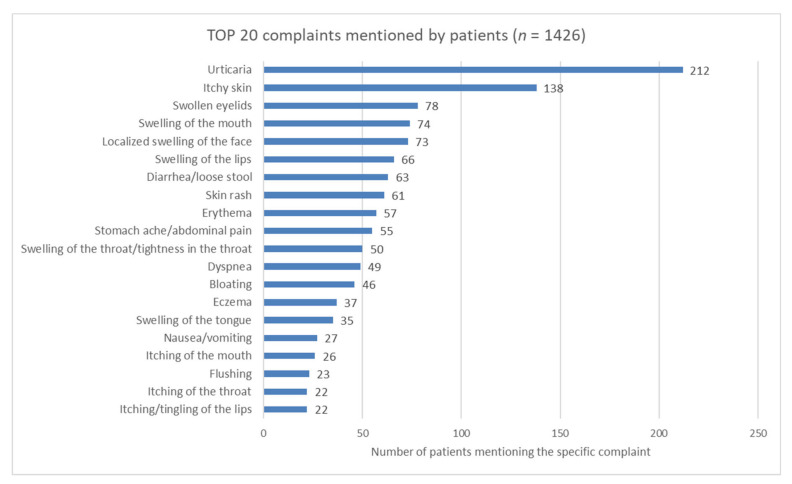
The 20 most commonly mentioned complaints in the patient group.

The most commonly reported complaints were urticaria (212/501, 42%) and generalized pruritus (138/501, 28%), followed by swollen eyelids (78/501, 16%), swelling of the mouth (15%) and lips (13%), and diarrhea/loose stool (13%) (Figure 2).

### 3.4. Referrals—Multidisciplinary, Complex Examination

**Figure 3:**
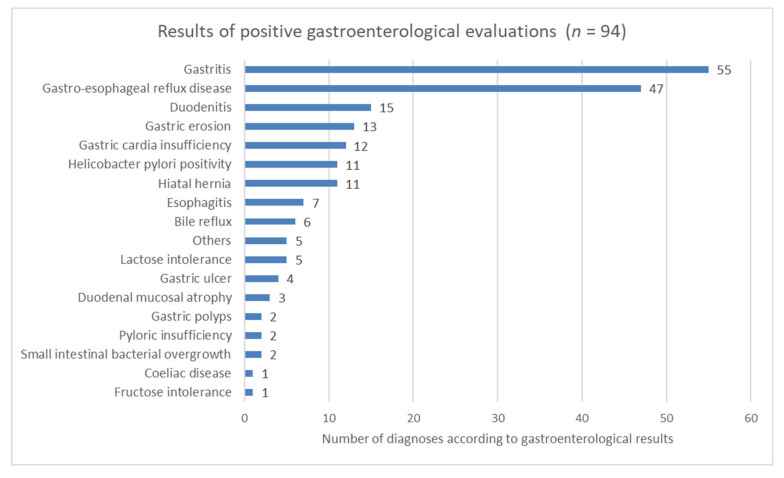
Results of positive gastroenterological examinations initiated as a result of the food-related complaints of patients.

Based on the listed complaints, possible diagnoses aside from food allergy emerged, so additional referrals and examinations were initiated (*n* = 1230) (Table 5). A gastroenterological examination was the most frequently required (320/501, 64%). Of the 320 referrals to the gastroenterologist, 113 results were returned (gastroenterological abnormalities were found in 94 cases; 19 patients returned negative results). The results of positive gastroenterological examinations initiated for food-related complaints show that gastritis and gastroesophageal reflux disease were the most commonly diagnosed diseases (Figure 3).

Referrals to other specialties (e.g., gynecology) or disciplines (dentistry) are not commonly considered in relation to allergies. However, in some cases chronic inflammation can occur latently, and often these are only exposed through certain dermatological complaints. Dental, ear-nose-throat, gynecological, and urological examinations were required (804/1230, 65%) due to symptoms suggestive of chronic inflammation.

In some cases, the possibility of a psychological background also arose. This manifested in sometimes bizarre suspected food/symptom associations (cheesecake-provoked speech acceleration and out-of-body feelings, chewing gum odor-provoked bloating, symptoms provoked by touching a chocolate pastry). Some complaints suggested aversion or avoidant/restrictive food intake disorder: one patient had no complaints related to tomatoes but felt unable to consume them raw (cooked tomatoes were non-problematic). Another patient said that beetroot “closed his throat” (the edematous mechanism was ruled out). Hand cramps while peeling asparagus was another complaint. Sometimes patients reported worsening skin symptoms through psychosocial stress. Anxiety disorders and panic attacks were also revealed by a detailed examination of some patients’ medical histories.

### 3.5. Dermatological Diagnoses

**Figure 4:**
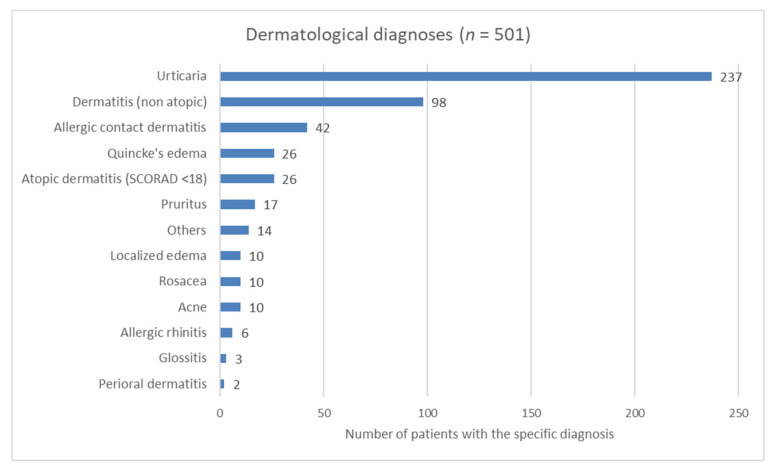
Dermatological diagnoses. SCORAD: SCORing Atopic Dermatitis.

Of the objectifiable dermatological diagnoses (Figure 4) based on medical history/photo documentation/present skin symptoms, urticaria (47%), dermatitis (20%), and allergic contact dermatitis (8%) were the most common.

Atopic predisposition (rhinitis, current or childhood asthma, current or childhood food allergy, and diagnosed atopic dermatitis) was detected among 168 patients (168/501, 34%).

### 3.6. Food-Related Diagnoses

**Figure 5:**
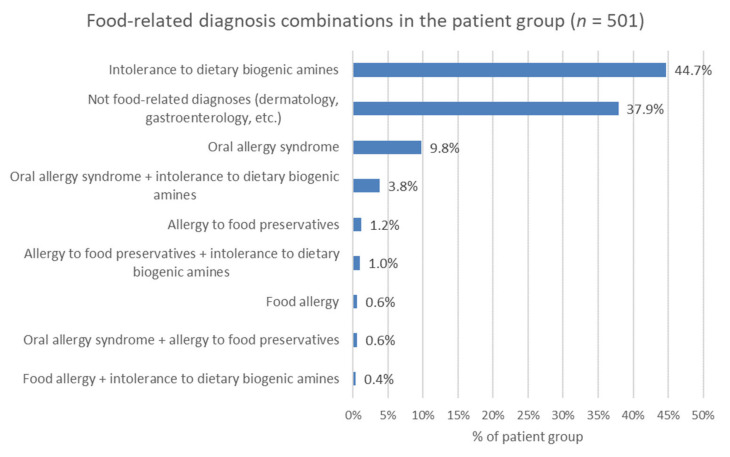
Food-related and non-food-related diagnosis combinations in patients with adverse food reactions.

Food-related diagnoses are shown in Figure 5. Intolerance to dietary biogenic amines occurred in 250 cases (250/501, 50%), in which patients received detailed spoken and written information about a diet low in dietary biogenic amines (mainly histamine and tyramine). The proportion of women and men diagnosed with intolerance to dietary biogenic amines was 46% (182/393) and 63% (68/108), respectively.

OAS was confirmed in 71 patients (71/501, 14%) based on medical history and inhalant allergen-specific IgE results. The proportions of women and men diagnosed with OAS were 13% (52/393) and 17% (19/108), respectively. The foods (raw vegetables, fruits, spices) that caused the complaint already indicated the problem in most cases.

Allergies to food preservatives were diagnosed in 14 (14/501, 3%) cases. Benzoic acid, sorbic acid, and sodium-disulfite positivity were observed. Sausages, cold cuts, mustard, croissants, bread, liver pâté, and instant soup caused itching, swelling of the mouth, and hives.

Five individuals (5/501, 1%) were diagnosed with IgE-mediated food allergy. All five patients had an atopic predisposition. Chicken eggs proved to be an allergen in three cases and milk in two. In one of the five cases, the food sensitivity (egg) had been previously known.

Patients were inclined to attribute a reaction to food even if it was caused by something else (e.g., a drug). In some cases (28/501, 6%), the edema-inducing/enhancing side effects of drugs (such as angiotensin-converting-enzyme inhibitors, angiotensin II receptor blockers, and beta blockers) had been misattributed by patients to various foods. In these cases, patients typically took the medicine with a meal; this could be the basis for the misunderstanding.

Toxic background occurred in two cases related to spoiled food. Considering the symptoms, carcinoid syndrome was suspected in two (2/501 0.4%) cases, while Melkersson–Rosenthal syndrome was suspected in one case (1/501, 0.2%).

### 3.7. Allergy Tests

Due to unconvincing medical histories from which food allergies could be ruled out with high certainty, allergy tests were not indicated in 37% (187/501) of patients. Food allergen-specific IgE tests were performed in 132 cases, inhalant allergen-specific IgE tests in 41 cases, and both in 141 cases. At least one food allergen positivity was detected in 54 cases, and from this group food allergy was found in five cases, consistent with the medical history, symptoms, and specific IgE positivity.

At least one inhalant allergen positivity was detected in 117 cases, and from this group OAS was found in 71 cases, consistent with medical history, symptoms, and specific IgE positivity.

## 4. Discussion

Data and other studies focusing on the frequency of actual food allergies and food intolerances among patients who consider themselves to be food allergic and therefore seek medical attention, as well as the examination of these patients, are lacking. The aim of this study was to describe patient-reported symptoms related to a suspected food allergy and to characterize and draw attention to the volume of patients who visit Budapest allergy clinics with a suspected food allergy. In this study, data were analyzed from patients who felt that their complaints were provoked by certain foods and whose severity of symptoms had already reached a level at which medical help was sought. Some patients had undertaken several therapeutic attempts at home without resolving their symptoms.

The results presented in this work reaffirmed that food allergies to classic allergens (a significant portion of which are due to the persistence of a childhood problem) are rare in adulthood, but arbitrarily restricted diets are common. OAS has also been shown to be frequent. The vast majority of patients diagnosed with OAS only experienced complaints during meals and were unaware that they had a pollen allergy or did not consider it significant relative to their food-associated complaints. The majority of complaints were caused by intolerance reactions and typically were complicated by gastroenterological diseases. In this study, reactions to dietary biogenic amines (e.g., histamine, tyramine) provoked the most symptoms. It is important to highlight that specific IgE tests do not always correlate with symptoms; their relevance should always be assessed individually and in conjunction with a detailed medical history.

The gap between the public assessment of food allergies and the opinions of physicians has been observed worldwide. In Thailand, the prevalence of food allergies reported by parents decreased from 9.3% to 1.11% after confirmation by a physician [37]. A similar trend was observed in Australia: the prevalence of self-reported food allergy (5.5%) was higher than doctor-confirmed cases (4.5%) [38]. In addition, a systematic review confirmed that the correlation between self-reported and challenge-confirmed food allergies is weak [6].

The risks of self-reported food allergies are the lack of proper examination, diagnosis, and treatment. As patients may tend to attribute a wide range of complaints to food allergies, the early symptoms of many diseases could remain hidden. Although the risk of over-restricted diets in childhood is more pronounced, adults—especially elderly patients—could be affected in several respects as well (e.g., malnutrition and its consequences) [32,39,40,41,42].

An interesting aspect of this issue is the low likelihood of medical advice being sought for food-related complaints. When asked, just 14.1–17.7% of participants said that they would consult a doctor for their complaints [43]. An online survey on the topic of self-reported food allergies found that almost half of the participants did not seek medical advice, while 31.4% sought the advice of an allergist consultant [44]. It is not clear why adults choose to limit their diets without consulting a doctor. It is possible that there are positive socio-economic effects that eliminate the motivation for a definitive diagnosis (whether confirmatory or not). A diagnosis that contradicts the patient’s previous opinion and lifestyle may not be desirable. This case scenario is likely, especially considering the low prevalence of adulthood food allergies; a patient will typically visit several doctors or clinics, hoping that the previous expert opinion that excluded food allergy from the diagnostic range was inaccurate [45]. Because these visits can strain the health care system, the simple yet effective, low-cost standardized diet history screening tool advised by Skypala et al. [18] would be beneficial for workers in primary care settings for simplifying distinguishing cases where food allergy is a real possibility from cases where it is not [46].

Conlon et al. [47] reviewed the types of allergy referrals and diagnostic outcomes received by an Irish allergy clinic. The main referral concern was food allergy (71% of cases). Although the study did not exclusively include patients with a self-suspected food allergy, it nonetheless showed the greatest similarity to the present work in regard to mean age (40 vs. 44) and male:female ratio (1:3.3 vs. 1:3.6). In both studies, the main presenting feature and the most common diagnostic outcome was urticaria/angioedema. In Conlon’s study, primary care accounted for 62% of the referrals, as opposed to just 30% for secondary care. In a Hungarian health care setting, the ratio between primary and secondary care referrals was more balanced at 43% and 53%, respectively.

The most common type of intolerance in this cohort was dietary biogenic amine intolerance (50%). The diagnosis of intolerance to dietary biogenic amines is entirely clinical [35] until a validated test system is available. The high proportion of cases may therefore be the result of unspecific diagnostic criteria and methods. Biogenic amines are vasoactive, low molecular weight substances. These include—but are not limited to—histamine, serotonin, dopamine, phenylethylamine, and tyramine (especially when taken in combination with monoamine oxidase inhibitors). They occur naturally in fermented products (sauerkraut, fish, cheeses, etc.). The presumed mechanism of the intolerance reaction is based either on the insufficient function of the degrading enzyme diamino-oxidase, or on the disproportion between enzyme capacity and the amount of substrate ingested. However, it should be noted that as stated in the “German guideline for the management of adverse reactions to ingested histamine”, “the scientific evidence to support the postulated link between ingestion of histamine and adverse reactions is limited, and a reliable laboratory test for objective diagnosis is lacking” [35]. Symptoms (erythema, urticaria, nausea, vomiting, diarrhea, abdominal pain) often present a varied picture similar to that of allergies. However, unlike with food allergies, the severity of complaints is directly proportional to the amount of biogenic amine-rich food consumed. Although dietary biogenic amines play a controversial role in triggering symptoms, our patients have reported improvements in skin and gastrointestinal condition when following a low biogenic amine diet. Patients often find it difficult to avoid foods high in biogenic amines and can therefore benefit from a dietary consultation [1,48,49].

A particular challenge in physician–patient communication was experienced. It was often difficult for patients to accept that nutritive-specific IgE tests alone only demonstrate sensitization, and without clinical reactions to the specific food do not provide evidence for the diagnosis of a food allergy. Additionally, some patients were reluctant to accept that, despite the positive result (IgE sensitization), further investigation was needed, as complaints may have been caused by other diseases with non-allergic mechanisms. The most common such diseases were intolerances (biogenic amine/histamine); gastroenterological diseases (reflux, gastritis, *Helicobacter pylori* colonization, chronic cholecystitis—gallstones); and dental, laryngeal, gynecological, or urological chronic inflammations.

Gastroenterological examination was most commonly required (64%), consistent with the high percentage of gastrointestinal complaints—42% of patients had some type of gastrointestinal disorder. Because patients were tested at a dermatology-allergology clinic, the available testing options were limited. Gastroenterological diseases such as eosinophilic esophagitis were diagnosed and treated by gastroenterologist consultants. Eosinophilic esophagitis is a condition of the esophagus with infiltration of eosinophils, which can be accompanied by heartburn, difficulty swallowing, and even nasal congestion, nausea, vomiting, and weight loss. It is closely related to atopic diseases (asthma, allergic rhinitis, eczema). The affected population is characterized by little or no response to anti-reflux medications. Corticosteroids, the endoscopic splitting of esophageal strictures, and diet have their place in the therapy of the disease, although the validity of the latter is disputed due to the sometimes conflicting results. The entire pathogenesis of eosinophilic esophagitis is not yet clear, but tissue expression of eotaxin-3 is considered important. Research also agrees that it is worth raising the possibility of food allergies, as diets based on the elimination of elementary or four to six typical allergens have brought significant improvements, especially in the histological picture (less in complaints), although omitted foods are not always consistent with serum-specific IgE positivity. The pathogenic role of minor allergens has also been raised. These cannot be detected by traditional methods, but only by molecular diagnostics [50,51]. It should be noted that eosinophilic esophagitis has also been reported as a side effect of oral or sublingual immunotherapy, therefore monitoring is required in these cases [52,53,54].

It is worth mentioning food protein-induced enterocolitis syndrome within the scope of non-IgE mediated reaction to foods with predominantly gastroenterological symptoms (repetitive vomiting, diarrhea, abdominal pain). It mainly affects infants and young children, although an adult form has also been described, the symptoms of which are triggered by various foods such as seafood and chicken eggs. The diagnosis is clinical and is based on the association between the culprit food and typical symptoms and on the resolution of symptoms after the elimination of symptom-provoking foods from the diet [55,56].

To the authors’ knowledge, this was the first large-scale prospective study to collect data on the characteristics of a patient population seeking specialist medical help for perceived/self-reported food allergies. In addition, this is the first study to assess the patterns of referrals made to an adult allergy clinic in Hungary due to adverse food reactions. Another way in which this study may contribute to the understanding of patients presenting with adverse food reactions is that, in all cases, a medical consultation was performed, followed—if appropriate—by a serum-specific IgE test. The prevalence data presented here were therefore physician-confirmed. Based on the results of three studies performed earlier at the Allergology Outpatient Unit, the prevalence of IgE-mediated food allergy in adult patients who suspected food allergy in the background of their complaints was only 1.1%, 1.5%, and 2.4% [31,57,58].

Even though this study aimed to provide data that were as detailed as possible, this work is not without limitations. The cohort does not represent either the general Hungarian population or the patient population visiting allergy clinics with similar problems. Patients living in and near the capital (Budapest) may be overrepresented. DBPCFC could not be performed. This work could not be compared with studies using questionnaires to estimate the prevalence of self-reported adverse food reactions (food allergy, food intolerance) due to differences in the selection criteria and objectives.

Taking the results into account, educational material will be prepared for the Hungarian public that draws attention to the differences between allergies and intolerances and emphasizes that not only food allergies cause food-related symptoms. Although comprehensive improvements in the health care system would require further, more detailed studies examining a larger number of cases, the presentation of patient group characteristics was aimed at providing guidance for service development efforts and assistance to health care professionals working in primary and secondary care, as well as in nutrition science and dietetics, who regularly encounter the problem of the differential diagnosis of adverse food reactions. In addition to the algorithm developed in the following phase of the study, the authors aim to develop one for the Hungarian population as an aid for practitioners and specialists. Current data enable us to outline a base for this algorithm, and its refinement will increase with cohort size.

In summary, it should be emphasized that the differential diagnosis of allergic and non-allergic conditions and the distinction between perceived and actual food allergies—as well as allergies and intolerances—remains a vital role for allergy clinics. The rationalization of patient management protocols would be an important step in ensuring that patients receive a timely diagnosis and do not wander within the health care system. Improvement is also needed in food allergy, intolerance, and general nutritional knowledge among the general public. It should be emphasized that, although a self-prescribed allergen-free diet without medical consultation is the solution preferred by some patients, it is not advisable to continue with ad hoc “free-from” meals or to avoid medical and dietetic specialist care. If a dietary restriction is unavoidable, the diet should be restricted only to the necessary extent and duration. Involving a dietitian in the nutrition care process is advisable. Further research into food avoidance and restriction in adults would be a useful contribution to developing an understanding of patients with adverse food reactions. Although not demonstrated in large-scale studies with control groups, histamine intolerance plays a notable role in non-immunological adverse food reactions. Distinguishing between histamine intolerance, other non-immune-mediated reactions, idiopathic reactions, organic diseases, adverse effects of medications, and psychosomatic reaction is essential for differential diagnosis.

## Figures and Tables

**Table 1:** Food and inhalant allergens tested [33].

Food Allergens	Inhalant Allergens
Rosacea:	Plants:
Apple	Birch pollen
Peach	Alder pollen
Tropical fruits:	Hazel pollen
Citrus mix *	Cottonwood pollen
Banana	Bermuda grass pollen
Vegetables:	Grass Mix ***
Carrot	Rye pollen
Celery	Mugwort pollen
Tomato **	Plantain pollen
Legumes:	Wall Pellitory pollen
Soybean	Goldenrod pollen
Peanut	Ragweed/Giant Ragweed pollen
Cereals:	
Rye flour	Fungi:
Wheat flour	Aspergillus fumigatus
Oilseeds:	Cladosporium herbarum
Almond	Alternaria alternata
Hazelnut	
Pumpkin	Animals:
Sesame	Dermatophagoides pteronyssinus
Animal allergens:	Dermatophagoides farinae
Casein ^##^	Feather Mix ^#^
Cow’s milk	Dog epithelia
Egg yolk	Cat epithelia
Egg white	
Codfish	

* Lemon, lime, orange, mandarin. ** Vegetable according to culinary classification [34]. *** Pollen of cocksfoot, meadow fescue, rye grass, Timothy grass, Kentucky bluegrass, velvet grass. ^#^ Feathers of goose, chicken, duck, turkey. ^##^ Allergen name: nBos d8.

**Table 2:** Common symptoms of biogenic amine intolerance [1,35,36].

Common Symptoms of Biogenic Amine Intolerance	
Skin	itching, flush reaction
Gastrointestinal tract	nausea, vomiting, diarrhea, abdominal pain
Cardiovascular system	tachycardia, sudden drop in blood pressure, dizziness
Respiratory system	rhinitis, sneezing, dyspnea

**Table 3:** Recommended foods and foods to avoid during a diet low in biogenic amines.

**Recommended Foods**
bread and pasta
potato, rice
steamed vegetables (except cabbage, spinach, tomato, peppers, beans, lentils, mushroom)
sausages, hams
meat: cooked, fried (excluding roasted and fried meat)
jams, fruit preserves, compotes
dairy products (butter, cottage cheese, sour cream, kefir, yoghurt, hard cheeses, etc.)
tea
water
**Not Recommended Foods**
walnut, poppy seed, hazelnut, oil seeds
cocoa, chocolate
peppers (paprika as well)
fish and seafood
smoked meat products
innards (offal)
eggs (boiled, fried-scrambled eggs, crumbed food)
cheese (camembert type spreadable cheeses)
citrus fruits, banana
alcohol (mainly red wine, beer)
coffee
sauerkraut
raw fruits and vegetables (paprika, tomato, onion, radish, cabbage)

**Table 4:** Demographic details and referral characteristics.

Patient Demographics	*n* = 501
Sex	
Female	393 (78%)
Male	108 (22%)
Male:female ratio	108:393 (1:3.6)
**Mean age, years (range, years)**	44 (18–85)
Age (years):	
18–29	119
30–39	101
40–49	99
50–59	67
60–69	83
70–79	29
80–89	3
Referral from:	
General practitioners	216 (43%)
Dermatologists	168 (34%)
Other specialists	96 (19%)
Others	21 (4%)

**Table 5:** Further referrals and examinations needed according to patients’ symptoms.

Specialist Consultation	Number of Referrals *
Gastroenterology	320
Abdominal ultrasound	120
Ear-nose-throat	176
Dentistry	184
Gynecology	114
Thyroid function test/ultrasound	110
Urology	33
Routine blood tests (inflammation!)	113
Clinical urine tests	60

* A patient may have multiple referrals.

## Abbreviations

α-Gal	Galactose-alpha-1,3-galactose
DBPCFC	Double-Blind Placebo-Controlled Food Challenge
OAS	Oral Allergy Syndrome
IgE	Immunoglobulin E
